# Inflammation-induced DNA methylation of DNA polymerase gamma alters the metabolic profile of colon tumors

**DOI:** 10.1186/s40170-018-0182-7

**Published:** 2018-07-10

**Authors:** Ashley R. Maiuri, Hongde Li, Barry D. Stein, Jason M. Tennessen, Heather M. O’Hagan

**Affiliations:** 1 Medical Sciences, Indiana University School of Medicine, Bloomington, Indiana USA; 2 Department of Biology, Indiana University, Bloomington, Indiana USA; 3 Indiana University Melvin and Bren Simon Cancer Center, Indianapolis, Indiana USA

**Keywords:** Inflammation, DNA methylation, DNA polymerase gamma, Mitochondria, Glucose uptake, Glycolysis

## Abstract

**Background:**

Inflammation, metabolism, and epigenetic modulation are highly interconnected processes that can be altered during tumorigenesis. However, because of the complexity of these interactions, direct cause and effect during tumorigenesis have been difficult to prove. Previously, using a murine model of inflammation-induced colon tumorigenesis, we determined that the promoter of the catalytic subunit of DNA polymerase gamma (*Polg*) is DNA hypermethylated and silenced in inflammation-induced tumors, but not in non-inflammation-induced (mock) tumors, suggesting that inflammation can induce silencing of *Polg* through promoting DNA methylation during tumorigenesis. *Polg* is the only mitochondrial DNA polymerase and mutations in *Polg* cause mitochondrial diseases in humans. Because of the role of mitochondria in metabolism, we hypothesized that silencing of *Polg* in inflammation-induced tumors would result in these tumors having altered metabolism in comparison to mock tumors.

**Methods:**

Inflammation-induced and mock colon tumors and colon epithelium from a mouse model of inflammation-induced colon tumorigenesis were assayed for alterations in *Polg* expression, mitochondria, and metabolism. Organoids derived from these tissues were used to study the direct effect of loss of *Polg* on mitochondria and metabolism.

**Results:**

We demonstrate that inflammation-induced tumors with reduced *Polg* expression have decreased mitochondrial DNA content and numbers of mitochondria compared to normal epithelium or mock tumors. Tumoroids derived from mock and inflammation-induced tumors retained key characteristics of the original tumors. Inflammation-induced tumoroids had increased glucose uptake and lactate secretion relative to mock tumoroids. shRNA-mediated knockdown of *Polg* in mock tumoroids reduced mtDNA content, increased glucose uptake and lactate secretion, and made the tumoroids more resistant to oxidative stress.

**Conclusions:**

These results suggest that inflammation-induced DNA methylation and silencing of *Polg* plays an important role in the tumorigenesis process by resulting in reduced mitochondria levels and altered metabolism. An enhanced understanding of how metabolism is altered in and drives inflammation-induced tumorigenesis will provide potential therapeutic targets.

**Electronic supplementary material:**

The online version of this article (10.1186/s40170-018-0182-7) contains supplementary material, which is available to authorized users.

## Background

Inflammation is known to directly contribute to epigenetic and metabolic alterations as well as tumor formation [1]. Furthermore, epigenetics and metabolism affect each other [2, 3]. For example, epigenetic alterations, including DNA methylation and histone modification, can result in heritable changes in expression of genes involved in metabolism without altering the DNA sequence of these genes. Additionally, alterations in metabolism can affect the availability of metabolites that act as cofactors or inhibitors of epigenetic enzymes.

Metabolism differs in cancer cells compared to normal cells with one of these changes being an increase in glycolysis [4]. This change in metabolism in cancer cells supports their higher proliferative rate and increased need to generate macromolecules for accelerated mitosis. One hallmark of increased glycolysis is an increase in glycolytic flux, which allows cancer cells to take up high levels of glucose even in the presence of physiological levels of ATP [5].

Mitochondria produce ATP through oxidative phosphorylation (OXPHOS) and in the process generate reactive oxygen species (ROS), redox molecules, and metabolites. They can sense cellular stress and assist cells in adapting to the environment making them important to the function of both normal and cancer cells. Because of their multiple cellular functions, the role of mitochondria in carcinogenesis is complex and can vary between cancers. While some cancers depend on mitochondrial respiration, other cancers have dysregulated mitochondria resulting in less efficient OXPHOS [6]. DNA polymerase γ (POLG) is the only mitochondrial DNA polymerase and is involved in the replication and repair of mitochondrial DNA (mtDNA). Furthermore, POLG is the only DNA polymerase capable of maintaining mitochondrial numbers [7]. POLG is a heterodimeric enzyme consisting of the catalytic core encoded by the *POLG* gene and an accessory subunit encoded by *POLG2*. Mutations in *POLG* result in reduced mitochondria and mitochondrial disorders in humans [8]. Experimentally reducing *POLG* expression levels results in decreased mtDNA levels [9]. Each mitochondrion contains multiple copies of mtDNA, and the copy number changes in response to energy demands. Expression of *POLG* and mtDNA copy number are controlled during development with high energy-consuming cells having a higher mtDNA copy number than lower energy cells [10]. DNA methylation of the promoter CpG island of *POLG* has been associated with reduced gene expression and decreased mtDNA copy number in highly proliferative versus terminally differentiated cells and several cancer cell lines [10, 11]. While DNA hypermethylation of *Polg* in human colorectal cancer has not been specifically studied, data from The Cancer Genome Atlas (TCGA) suggests that some colorectal cancers have increased levels of *Polg* promoter DNA methylation [12]. Furthermore, mutations in *POLG* have been found in human breast cancers and are associated with decreased mtDNA levels and decreased mitochondrial activity [13].

We use a mouse model of inflammation-induced tumorigenesis to study the initiation of DNA methylation by inflammation. In this model, mice are infected with a human commensal bacterium, enterotoxigenic *Bacteroides fragilis* (ETBF). ETBF infection induces acute followed by chronic inflammation in the distal colon [14]. Infection of multiple intestinal neoplasia (Min) mice, which are heterozygous for loss of the tumor suppressor gene *adenomatous polyposis* (*Apc*), results in Th17 inflammation-driven tumorigenesis in the distal colon [15]. ETBF-infected Min mice develop on average 13 distal colon tumors (ETBF Min tumors) as compared to mock-infected mice that only occasionally develop colon tumors (Mock Min tumors––0.3 tumors per mouse) [16]. Using this model, we have demonstrated that inflammation-induced tumors have DNA hypermethylated regions relative to epithelium from mock-infected mice (mock Min epithelium) that are not present in non-inflammation-induced tumors from mock-infected mice (mock Min tumors) [16]. Inflamed epithelium isolated from ETBF-infected mice after the removal of tumors (ETBF Min epithelium) has an intermediate level of DNA hypermethylation at regions altered in ETBF Min tumors. Importantly, we demonstrated that the initiation of epigenetic changes is dependent on the mismatch repair protein MSH2 (MutS homolog 2) recruiting epigenetic proteins to sites of inflammation-induced oxidative damage. Mice lacking MSH2 in their intestinal epithelial cells (*Msh2*^*l/l*^*VC*Min) are deficient in mismatch repair, and ETBF infection of these mice results in an increase in tumorigenesis that is localized to the same region of the colon as in Min mice (a mean of 30 distal colon tumors per mouse) [16]. Interestingly, these tumors (ETBF *Msh2*^*l/l*^*VC*Min tumors) have very few regions with DNA hypermethylation and their genome-wide methylation profiles are more similar to mock Min tumors than ETBF Min tumors. *Polg* was one of the genes found to be specifically silenced through promoter CpG-island DNA hypermethylation in inflammation-induced tumors with wildtype MSH2, and a lack of MSH2 in intestinal epithelial cells prevented *Polg* methylation and loss of expression in inflammation-induced tumors [16].

Because of POLG’s role in mitochondrial function, we hypothesized that reduced expression of *Polg* would result in reduced mtDNA and altered metabolism in inflammation-induced tumors as compared to non-inflammation-induced tumors. This study reports a novel functional role for *Polg* methylation during inflammation-driven tumorigenesis, specifically a reduction in mitochondria that is correlated with an increase in glucose uptake and resistance to oxidative stress.

## Methods

### Animal model

Min^*Apc*Δ716±^ and *Msh2*^*l/l*^*VC*Min mice were handled and inoculated with ETBF as described previously [15, 16]. For all experiments, both males and females were used, mice were randomized between mock and ETBF groups, and mice of different genotypes were cohoused. Individual tumors were removed from dissected colons at 4 or 8 weeks post-infection with the aid of a dissecting microscope and stored at − 80 °C until further analysis. Distal (0–2 cm measured from the rectum) epithelium was collected by scraping the mucosal surface of the dissected colon (after removal of any tumors), washed three times in PBS, and then subjected to the indicated protocol. Such scraping has been shown to be an effective method to obtain samples of intestinal epithelial cells [17].

### Quantitative methylation-specific PCR (qMSP) and mtDNA content

Isolated DNA (QIAamp DNA Micro Kit, Qiagen) was bisulfite-treated (EZ DNA methylation-Gold kit, Zymo Research), and qMSP assays were performed as described previously [16]. Quantification of mtDNA levels relative to nuclear DNA (ApoB– Apolipoprotein B) was performed using qPCR and the delta Ct method [18, 19]. See Additional file 1: Table S1 for primers used.

### Gene expression

RNA was prepared from epithelium, tumors, or organoids using Trizol followed by cleanup with a RNeasy kit (Qiagen). cDNA was prepared, and qPCR was done using TaqMan assays (see Additional file 1: Table S1 for assays used). Expression of candidate genes was normalized to expression of a housekeeping gene (*PPIA*).

### Transmission electron microscopy

Four weeks post-infection colons were dissected, opened, and rinsed with PBS. A 1-mm section was cut from the most distal 0–0.5 cm region and fixed on ice in 2.5% glutaraldehyde, 4% paraformaldehyde, 0.1 M sodium cacodylate, and pH 7.2 for 16 h. While on ice, the samples were processed further by being put into four changes of buffer to remove fixative, followed by 2% osmium tetroxide in 0.1 M sodium cacodylate buffer, pH 7.2 for 2 h, two changes of buffer to remove residual osmium tetroxide, and through a graded ethanol dehydration series to 100% ethanol. At room temperature, the samples were placed in three changes of 100% ethanol, 2:1, 1:1, 1:2 of ethanol/propylene oxide, 100% propylene oxide, infiltrated first with 1:1, 1:3, 1:6 propylene oxide/Embed 812 resin (Electron Microscopy Sciences, Hatfield, PA), and then with three changes of 100% resin before polymerization at 65 °C for 18 h. The polymerized blocks were sectioned at 70 nm thickness using a Leica Ultracut UCT ultramicrotome. Sections were placed on 300-mesh copper grids. The grids were stained first with saturated uranyl acetate, washed with water, and then with lead citrate. The grids were viewed with a JEOL company JEM-1010 transmission electron microscope and photographed using a Gatan 1 k × 1 k model 794 MultiScan camera. A single tumor was imaged per condition with five images taken per sample by a blinded investigator.

### Metabolomic analysis

Metabolomic analysis was performed using gas chromatography-mass spectrometry (GC-MS) as previously described with slight modifications [20]. Namely, ~ 5–10 mg of distal colon epithelium or tumors were washed twice in ice-cold PBS; then, metabolites were extracted with 800 μL of 90% cold methanol containing 2 μg/mL of succinic acid-2,2,3,3-d4 as an internal standard using the Omni Bead Ruptor 24 homogenizer (Omni International), and dried with a Speed-Vac. Dried extracts were derivatized with O-methoxylamine hydrochloride and *N*-methyl-*N*-trimethylsilyltrifluoracetamide containing 1% trimethylchlorosilane, respectively. Derivatized samples were analyzed in an Agilent 6890 GC-5973iMS equipment with a 30-m Phenomex ZB5-5 MSi column with a 5-m long guard column. Raw data were normalized with the internal standard and tissue mass.

### Organoids

Organoids were derived from mock distal colon epithelium (colonoids) or mock or inflammation-induced Min colon tumors (tumoroids) as demonstrated previously and grown in basal growth media containing EGF (epidermal growth factor), Noggin, R-Spondin1, and Wnt3a (colonoids) or EGF and Noggin (tumoroids) [21, 22]. shRNA-mediated *Polg* knockdown was performed by dissociating tumoroids into single cells using TrypLE, plating in Matrigel and incubating in media containing polybrene plus nontarget (NT) or *Polg*-concentrated lentivirus for 2 h. Then, the media were changed to normal tumoroid medium supplemented with 10 μM Y27632. Forty-eight hours later, media were replaced with fresh media containing 4 μg/ml puromycin. Knockdown organoids were passaged at least one time before performing downstream assays.

### Proliferation assay

Tumoroids were disassociated into single cells and 4000 cells in 25 μL Matrigel were plated per well of a 48-well plate. On the indicated day, tumoroids were lysed by homogenization in 200 μL Lactate Assay Buffer (BioVision). The DNA concentration of the lysate was then determined using the Qubit dsDNA HS assay (Invitrogen), which is highly selective for DNA over RNA. Readings were found to be proportional to but more sensitive than cell numbers (data not shown). A similarly treated well without any cells added was used as a blank for the readings. Total DNA content was calculated by multiplying the concentration of DNA by the total volume of buffer.

### Glucose uptake, glucose depletion, and lactate secretion

Glucose uptake assays were performed using the Glucose Uptake Colorimetric Assay Kit (BioVision) with some modifications. After 5 days of growth, tumoroids were incubated in 250 μL KRPH buffer (20 mM HEPES, 5 mM KH_2_PO_4_, 1 mM MgSO_4_, 1 mM CaCl_2_, 136 mM NaCl, 4.7 mM KCl, pH 7.4) for 40 min followed by the addition of 25 μL of 10 mM 2-DG (2-deoxy-D-glucose) and an additional 20-min incubation. Washed tumoroids were then removed from the Matrigel by pipetting up and down in cold PBS and lysed in extraction buffer. A 2-DG6P standard curve was performed during each experiment and used to calculate 2-DG uptake. As per the manufacturer’s recommendation, protein concentration (BCA) assays were performed on the same cell lysate from each sample that was used for the glucose uptake assay to determine the protein concentration, which was then used to normalize the 2-DG uptake.

The same wells of tumoroids were used for glucose depletion and lactate secretion assays. Media were collected from each tumoroid well at the indicated day post-plating and used immediately for the assays. Glucose levels were measured using 2 μL media and the Glucose (HK) Assay Kit (Sigma-Aldrich) following the manufacturer’s directions. Media from a similarly treated well without any cells added were used to determine the starting glucose levels. The glucose standard provided with the kit was used during each experiment to calculate glucose levels. Glucose depletion was calculated by subtracting the glucose levels in the media of a given well from the media from the no cells well. The concentration of DNA in each well was determined as in the “Proliferation assay” section and used to normalize the depleted glucose levels. To determine lactate levels, media were diluted 1:10 or 1:20 in lactate assay buffer and 2 μL was assayed with the Lactate Colorimetric/Fluorometric Assay Kit (BioVision) following the manufacture’s protocol. A L(+)-Lactate standard was assayed during each experiment and used to calculate lactate levels. Lactate levels were normalized to DNA concentration as above. For NT and Polg KD tumoroids, lactate levels in media of wells containing tumoroids were determined as above at day 5 post-plating.

### Tumoroid viability

Seventy-two hours after the addition of PBS or 0.5 mM H_2_O_2_ to the media of tumoroid cultures, the media were removed and 250 μL of KRPH buffer was added plus MTT (3-(4,5-dimethylthiazol-2-yl)-2,5-diphenyltetrazolium bromide) to a final concentration of 500 μg/ml and incubated at 37 °C for 1 h. Matrigel and MTT were solubilized as previously described [23].

### Statistical analysis

Expression data, qMSP, mtDNA content, metabolite levels, glucose uptake, and cell viability are presented as the mean ± standard error (SEM). Data are evaluated by Mann-Whitney *U* test and considered statistically significant with a *p* value < 0.05. Sample sizes are indicated in associated figure legends for assays using tumor tissue. For all assays performed with organoid experiments were performed three times with two to three biological replicates per experiment. The mean ± SEM is computed using all biological replicates from all experiments.

## Results

### DNA methylation of *Polg* is associated with reduced mtDNA content in inflammation-induced colon tumors

**Fig. 1:**
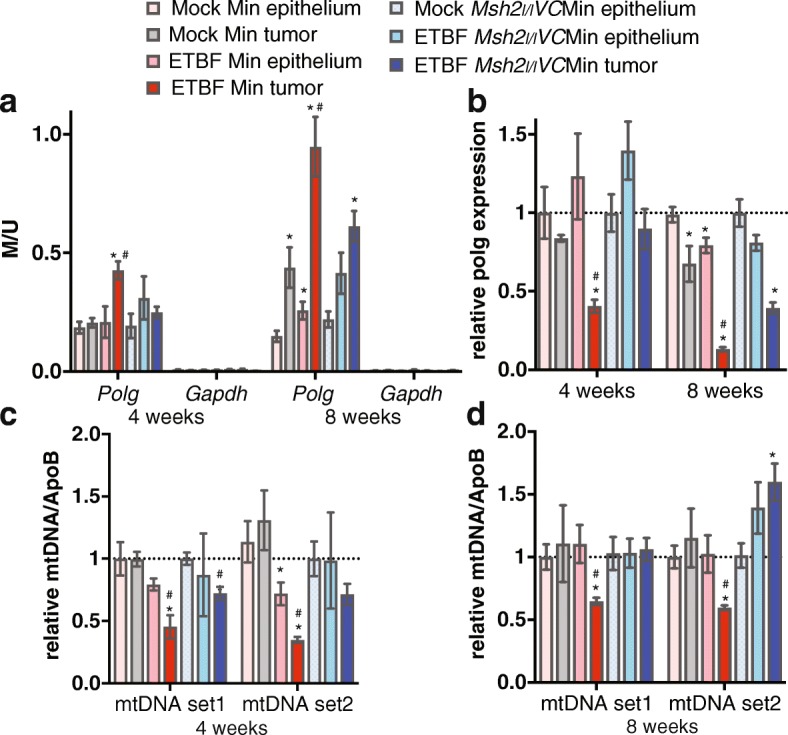
Tumors with promoter DNA methylation and reduced expression of *Polg* have reduced mitochondrial DNA content. **a** qMSP of bisulfite-treated DNA from indicated tissue 4 or 8 weeks post-infection using methylation-specific primers in promoter CpG islands of indicated genes. Mean ± SEM. *N* = 4–5 for epithelium, *N* = 5–9 for tumors. *P < 0.05 compared with mock epithelium. ^#^*P* < 0.05 compared to ETBF Min epithelium and mock Min and ETBF *Msh2*^*l/l*^*VC*Min tumors. **b** *Polg* gene expression by qPCR relative to mock epithelium. Mean ± SEM. *N* = 4–8 for epithelium, *N* = 6–12 for tumors, asterisk and number sign as in **a**. DNA from tissue (**c**) 4 weeks or (**d**) 8 weeks post-infection was used in qPCR assays with primer sets specific for two different regions of mitochondrial DNA or the genomic ApoB. Mean ± SEM. *N* = 4–5 for epithelium, *N* = 5–9 for tumors, asterisk and number sign as in **a**

Using methyl CpG-binding domain sequencing (MBD-seq), we previously found that inflammation-induced tumors (ETBF Min tumors) collected from Min mice 8 weeks post-ETBF infection have unique DNA hypermethylated regions that are mostly not hypermethylated in non-inflammation-induced tumors (mock Min tumors) [16]. Additionally, we demonstrated that in mice lacking expression of the mismatch repair protein MSH2 in their intestinal epithelium, the inflammation-induced DNA hypermethylation and expression alterations in tumors were reduced as compared to ETBF Min tumors [16]. The CpG-island containing promoter of *Polg* is one of regions specifically DNA hypermethylated in ETBF Min tumors. As POLG is the only mitochondrial DNA polymerase involved in the replication and repair of mitochondrial DNA, and mitochondria play a critical role in tumorigenesis, tumor progression, and metabolic regulation, we decided to explore the association between the DNA methylation of *Polg* and tumor metabolism. Tumors can be macrodissected as early as 4 weeks post-infection in our mouse model. Therefore, we assayed *Polg* DNA methylation in early tumors and epithelium from Min and *Msh2*^*l/l*^*VC*Min mice as well as 8-week tumors and epithelium for comparison. Four- and 8-week ETBF Min tumors had significantly higher levels of *Polg* DNA methylation than epithelium from mock-infected mice (mock Min epithelium) (Fig. 1a). In contrast, ETBF Min epithelium and mock Min and ETBF *Msh2*^*l/l*^*VC*Min tumors had increased *Polg* DNA methylation compared to mock Min epithelium 8 weeks post-mock infection, but not at 4 weeks, and this increase in DNA methylation was still significantly less than the *Polg* methylation in ETBF Min tumors. No significant changes in *Polg* promoter methylation were detected in inflamed epithelium from *Msh2*^*l/l*^*VC*Min mice (Fig. 1a). Lack of DNA methylation in the CpG island containing promoter of *Gapdh* in all samples serves as a negative control.

Promoter CpG-island DNA hypermethylation is known to be associated with reduced gene expression. *Polg* expression was decreased in 4- and 8-week ETBF Min tumors relative to mock and ETBF Min epithelium and mock Min and ETBF *Msh2*^*l/l*^*VC*Min tumors (Fig. 1b). Confirming the relationship between DNA methylation and expression, *Polg* expression in 8-week ETBF Min tumors was less than 4-week ETBF Min tumors. Additionally, *Polg* expression was only decreased in ETBF epithelium and mock Min and ETBF *Msh2*^*l/l*^*VC*Min tumors versus mock epithelium 8 weeks post-infection when there is a corresponding increase in DNA methylation. There was a trend towards reduced expression of *Polg* in ETBF *Msh2*^*l/l*^*VC*Min epithelium 8 weeks post-ETBF, but the change was not statistically significant (Fig. 1b).

Because reduced expression of *Polg* has been associated with reduced mtDNA content [9], we assayed mtDNA content relative to genomic DNA content in the various tissue types. At 4 and 8 weeks post-infection, ETBF Min tumors had significantly less mtDNA content than mock or ETBF Min epithelium or mock Min or ETBF *Msh2*^*l/l*^*VC*Min tumors (Fig. 1c, d). mtDNA content in mock Min tumors was not significantly different from the mock epithelium at either time point. At 4 weeks post-ETBF mtDNA content in ETBF *Msh2*^*l/l*^*VC*Min tumors was slightly reduced depending on the locus examined, but was unchanged or increased at the 8-week time point (Fig. 1d). mtDNA content in ETBF epithelium was also partially reduced at 4 weeks, but returned to normal levels at 8 weeks post-ETBF. Altogether, these data demonstrate that inflammation-induced tumors with *Polg* promoter CpG-island DNA hypermethylation have reduced *Polg* gene expression and mtDNA content.

### Inflammation-induced tumors with wildtype MSH2 have fewer mitochondria

**Fig. 2:**
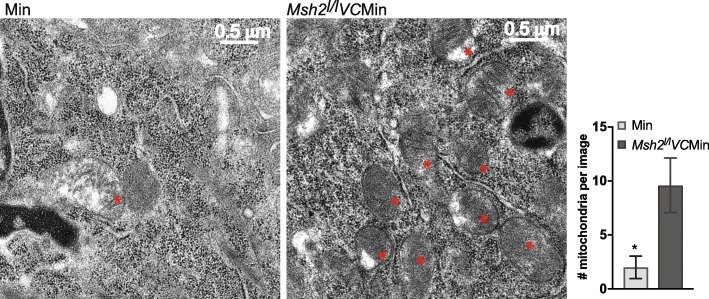
Inflammation-induced tumors with wildtype MSH2 have fewer mitochondria. One tumor each from mice of the indicated genotypes 4 weeks post-ETBF infection was imaged by transmission electron microscopy. Red asterisk indicates mitochondria. Five images at the same magnification were taken per sample in a blinded fashion, and the numbers of mitochondria per image were counted. Mean ± SEM. *P < 0.05

Mitochondria have multiple copies of mtDNA, and the number can vary between cell types [10], suggesting that the decrease in mtDNA content in the ETBF tumors could be from decreased number of mtDNA copies per mitochondria or decreased numbers of total mitochondria in the tumors. Therefore, we used transmission electron microscopy (TEM) to image mitochondria in tumors from our mouse model. Because of size limitations of TEM, we used tissues from mice 4 weeks post-infection when tumors are still relatively small. Since mitochondria can be relocalized inside cells in response to stress, it was important to use control tissue that was undergoing similar inflammation-induced stress [24]. Therefore, ETBF *Msh2*^*l/l*^*VC*Min tumors are the best comparison tissue for ETBF Min tumors. TEM images of ETBF Min tumors had fewer mitochondria than ETBF *Msh2*^*l/l*^*VC*Min tumors (2.0 versus 9.6 mitochondria per image, respectively) (Fig. 2). There were no obvious structural differences in the mitochondria that were present between the two different tumor types. This finding suggests that reduced expression of *Polg* by inflammation-induced DNA hypermethylation results in fewer mitochondria in tumors although some are still present and likely necessary for cell survival.

### Altered expression of metabolic genes and metabolite levels in inflammation-induced tumors

**Fig. 3:**
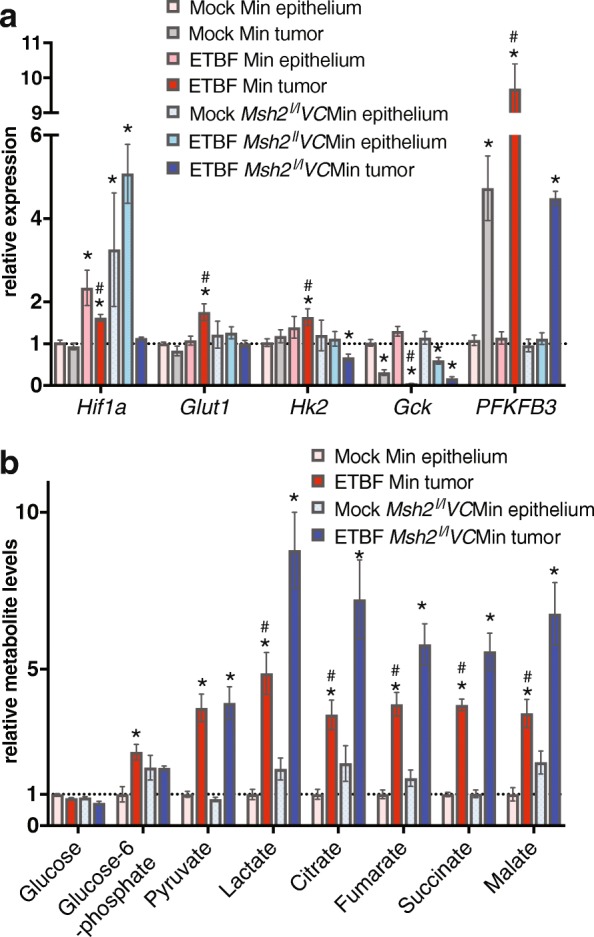
Inflammation-induced tumors have altered metabolism. **a** Gene expression by qPCR relative to mock Min epithelium from indicated tissue 8 weeks post-infection. Mean ± SEM. *N* = 4–8 for epithelium, *N* = 6–12 for tumors. *P < 0.05 compared with mock Min epithelium. ^#^*P* < 0.05 compared to mock Min and ETBF *Msh2*^*l/l*^*VC*Min tumors. **b** Metabolites in tissues as in **a**. Mean ± SEM. *N* = 4 tissue, six tumors. *P < 0.05 compared with mock epithelium. ^#^*P* < 0.05 compared to ETBF *Msh2*^*l/l*^*VC*Min tumors

Altered mitochondrial function has been associated with increased aerobic glycolysis [6]. To determine the alterations in metabolic gene expression in ETBF Min tumors versus mock Min epithelium, we first used a PCR array for detecting expression of genes involved in glucose metabolism as well as screened additional candidates by qPCR. Using a two-fold cutoff, three genes on the array had increased expression in inflammation-induced tumors (*Phosphoribosyl pyrophosphate synthetase 1-Like 1 Prps1l1*; *Hexokinase 3* - *Hk3*; *Glycogen phosphorylase, muscle associated* - *Pygm*) and 17 genes had decreased expression on the PCR array (Additional file 1: Table S2). We verified expression alterations of genes with the greatest fold change (*Glucokinase* –*Gck*; *Aldolase, fructose-bisphosphate B* – *Aldob*; *Prps1l1*; *Hk3*) by qRT-PCR, and all expression changes were validated (Fig. 3a and Additional file 1: Figure S1A). To determine which gene expression changes may be associated with reduced expression of *Polg* and reduced mitochondria, we also performed qRT-PCR using mock Min tumors and ETBF-induced *Msh2*^*l/l*^*VC*Min tumors, which have higher levels of *Polg* expression and mtDNA content than ETBF Min tumors. *Hif1a* (*Hypoxia inducible factor 1 alpha*), *Glut1* (*glucose transporter 1*), and *Hk2* had increased expression in ETBF Min tumors relative to mock Min epithelium as well as relative to mock Min and ETBF *Msh2*^*l/l*^*VC*Min tumors (Fig. 3a). None of these genes had significant increases in expression in the other tumor types relative to mock Min epithelium. *Hif1a* expression was also increased in mock *Msh2*^*l/l*^*VC*Min epithelium and ETBF epithelium from Min and *Msh2*^*l/l*^*VC*Min mice relative to mock Min epithelium. Expression of *6-phophofructose-2-kinas/fructose2,6-biphosphatase 3* (*Pfkfb3*), the only isoform of *Pfkb* that is induced by inflammatory stimuli and hypoxia, was significantly increased in all tumors assayed relative to mock Min epithelium and was significantly higher in ETBF Min tumors than mock Min or ETBF *Msh2*^*l/l*^*VC*Min tumors (Fig. 3a). *Pfkfb3* expression was not altered in ETBF epithelium from either genotype. *Gck* expression was reduced in all tumor types, but was significantly lower in ETBF Min tumors compared to the other tumor types (Fig. 3a). *Gck* expression was also decreased in ETBF *Msh2*^*l/l*^*VC*Min epithelium. Interestingly, all of the expression changes in ETBF Min tumors are associated with increased glycolytic flux [5, 25]. Stabilization of HIF1α protein and activation of AKT (AKT serine/threonine kinase 1) are common occurrences in tumors and both of them can alter the expression of metabolic genes [26]. However, by western blot, total HIF1α and phosphorylated AKT were elevated to similar levels in ETBF and mock Min tumors relative to mock Min epithelium (Additional file 1: Figure S1B).

To more directly determine if the inflammation-induced tumors have altered metabolism, we assayed metabolite levels in epithelium and tumors. We focused on inflammation-induced tumors from Min and *Msh2*^*l/l*^*VC*Min mice because these tumors were undergoing similar inflammation-induced stress but had different *Polg* and mtDNA levels. While there was no change in glucose levels, the levels of glucose-6-phosphate (G-6P) were higher in ETBF Min tumors than mock Min epithelium (Fig. 3b). G-6P levels in ETBF *Msh2*^*l/l*^*VC*Min tumors were unaltered relative to mock *Msh2*^*l/l*^*VC*Min epithelium. Pyruvate levels were increased similarly in ETBF Min and *Msh2*^*l/l*^*VC*Min tumors relative to their respective epithelium (Fig. 3b). Lactate and the tricarboxylic acid (TCA) metabolites (citrate, fumarate, succinate, malate) were elevated in both tumor types relative to their respective epithelium (Fig. 3b). However, levels of these metabolites were significantly higher in ETBF *Msh2*^*l/l*^*VC*Min tumors relative to ETBF Min tumors. Altogether, these data suggest that both types of tumors have altered metabolite levels relative to their respective mock epithelium, but that ETBF Min tumors have increased G-6P and lower mitochondrial metabolites than ETBF *Msh2*^*l/l*^*VC*Min tumors.

### Tumoroids derived from inflammation-induced tumors have increased glucose uptake

**Fig. 4:**
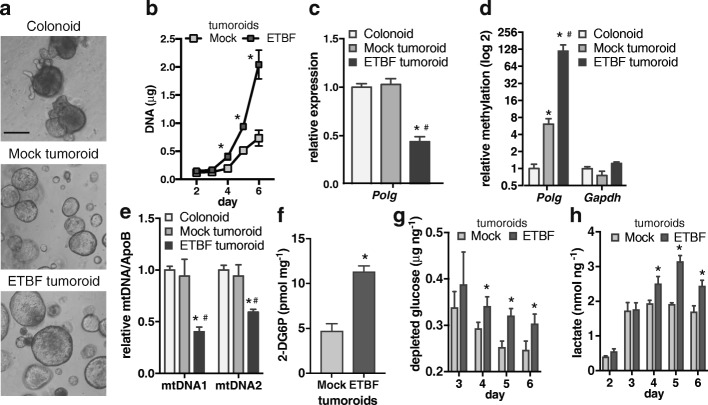
Tumoroids derived from inflammation-induced tumors have increased glucose uptake. **a** Representative images of colonoids derived from mock Min epithelium and tumoroids derived from mock or ETBF Min tumors 8 weeks post-infection. Scale bar, 100 μm. **b** Total DNA content in wells of mock and ETBF tumoroids as in **a** collected at the indicated days post-plating. Mean ± SEM. The data is from three independent experiments each with two biological replicates. *P < 0.05. **c** *Polg* gene expression in organoids as in **a** by qPCR relative to epithelium organoids. Mean ± SEM. The data is from three independent experiments each with two biological replicates. *P < 0.05 compared to epithelium. ^#^*P* < 0.05 compared to mock tumoroid. **d** qMSP of bisulfite-treated DNA from organoids as in **a** using methylation-specific primers in promoter CpG islands of indicated genes. Bar indicates mean ± SEM. The data is from three independent experiments each with two biological replicates, asterisk and number sign as in **c**. **e** DNA from organoids as in **a** was used in qPCR assays with primer sets specific for two different regions of mitochondrial DNA or the genomic ApoB. Mean ± SEM. The data is from three independent experiments each with two biological replicates, asterisk and number sign as in **c**. **f** Glucose uptake by tumoroids derived as in **a**. 2-DG6P levels were normalized to protein concentrations of the samples to account for differences in cell number. Mean ± SEM. The data is from three independent experiments each with three biological replicates. *P < 0.05. **g** At the indicated day post-plating, glucose levels in media from wells with tumoroids were subtracted from glucose levels in media from a well with Matrigel, but without cells, and then normalized to DNA concentration of the tumoroids. Mean ± SEM. The data is from three independent experiments each with two biological replicates. *P < 0.05. **h** Lactate levels in media from wells with tumoroids at the indicated day post-plating normalized to DNA concentration. Mean ± SEM. The data is from three independent experiments each with two biological replicates. *P < 0.05

The increased expression of genes related to glycolytic flux as well as increased G-6P metabolite levels suggest that inflammation-induced tumors have increased glucose uptake. To be able to directly assay glucose uptake in tumor cells, we derived organoids from tumors from mock or ETBF-infected Min mice (tumoroids) as well as organoids from mock colon epithelium (colonoids). Since tumoroids and colonoids only consist of tumor or epithelial cells, respectively, they provide a reductionist approach to examining cellular changes without other cells that are normally present in the mucosal layer of the colon [27]. Colonoids formed characteristic budding structures, whereas mock and ETBF tumoroids formed cystic structures that were similar in appearance to each other (Fig. 4a). ETBF tumoroids proliferated significantly faster than mock tumoroids as demonstrated by increased DNA content per well of tumoroids at days 4, 5, and 6 post-plating for ETBF relative to mock tumoroids (Fig. 4b). We verified that ETBF tumoroids maintain reduced *Polg* expression relative to colonoids and mock tumoroids (Fig. 4c). We then verified that ETBF tumoroids have the increased *Polg* promoter CpG-island methylation present in ETBF Min tumors by performing qMSP on isolated DNA. ETBF tumoroids did have higher levels of DNA methylation at the *Polg* promoter than colonoids and mock tumoroids, but not at the *Gapdh* promoter, which does not undergo inflammation-induced DNA hypermethylation (Fig. 4d). Furthermore, ETBF tumoroids had lower levels of mtDNA than colonoids and mock tumoroids consistent with our previous data (Fig. 4e). Interestingly, ETBF tumoroids had increased glucose uptake relative to mock tumoroids (Fig. 4f; measured 5 days after plating). To confirm these results by a different assay, glucose levels in the media that the tumoroids were growing in were measured and normalized to the DNA content of the tumoroids. ETBF tumoroids depleted the media of more glucose than mock tumoroids at days 4, 5, and 6 post-plating (Fig. 4g). Since aerobic glycolysis results in increased production and secretion of lactate, we measured lactate levels in the media and normalized these values to DNA content. Lactate secretion by ETBF tumoroids was more than mock tumoroids at days 4, 5, and 6 post-plating (Fig. 4h). Changes in glucose levels in the media were undetectable at 2 days post-plating, and glucose and lactate levels were not different between the tumoroid types at 3 days post-plating. These findings demonstrate that epigenetic changes persist in tumoroids and that tumoroids derived from inflammation-induced tumors with reduced *Polg* expression have increased proliferation, glucose uptake, and lactate secretion than mock tumoroids.

### Reduction of Polg expression increases glucose uptake and resistance to oxidative stress in mock tumoroids

Tumors have many epigenetic and other types of alterations compared to normal epithelium. To determine if reduced *Polg* expression directly affects glucose uptake, we first knocked down *Polg* in colonoids derived from the epithelium of WT-uninfected mice. shRNA knockdown of *Polg* resulted in decreased *Polg* expression and reduced mtDNA (Additional file 1: Figure S2A&B). However, glucose uptake was similar in nontarget (NT) and *Polg* knockdown (KD) colonoids suggesting that reduced expression of *Polg* in WT colonoids is not sufficient to alter glucose uptake (Additional file 1: Figure S2C).

**Fig. 5:**
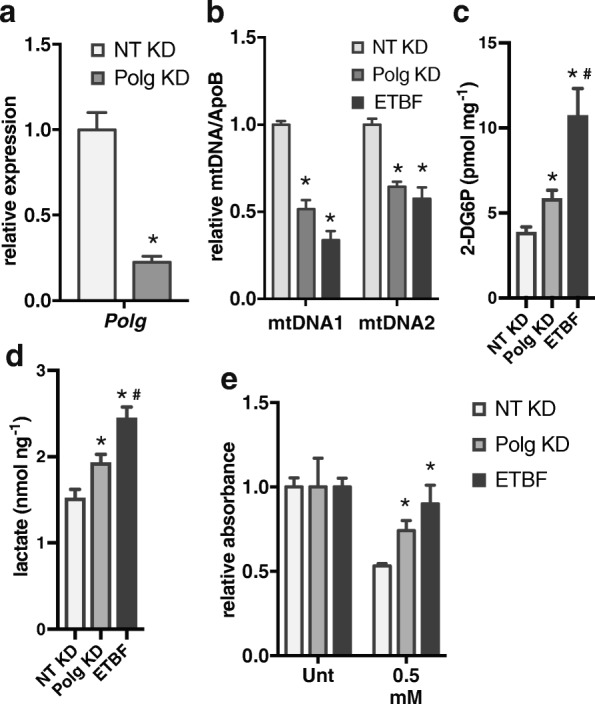
Reduced *Polg* expression increases glucose uptake and resistance to oxidative stress. **a** *Polg* gene expression in nontarget (NT) or *Polg* knockdown (KD) mock tumoroids by qPCR relative to NT organoids. Mean ± SEM. The data is from three independent experiments each with two biological replicates. *P < 0.05. **b** DNA from tumoroids as in **a** was used in qPCR assays with primer sets specific for two different regions of mitochondrial DNA or the genomic ApoB. Mean ± SEM. The data is from three independent experiments each with two biological replicates. *P < 0.05 compared to NT. **c** Glucose uptake by tumoroids derived as in **a** normalized to protein concentration. Mean ± SEM. The data is from three independent experiments each with three biological replicates. *P < 0.05 compared to NT. ^#^*P* < 0.05 compared to Polg KD. **d** Lactate levels in media from wells with tumoroids as in **a** normalized to DNA concentration. Mean ± SEM. The data is from three independent experiments each with two biological replicates, asterisk and number sign as in **c**. **e** Cell viability relative to untreated assayed by MTT in tumoroids derived as in **a** 72 h after mock treatment (Unt) or 0.5 mM H_2_O_2_. Mean ± SEM. The data is from three independent experiments each with two biological replicates. *P < 0.05 compared to NT

To determine if reduced *Polg* expression is sufficient to alter metabolism in tumor cells, we knocked down *Polg* in mock tumoroids. *Polg* KD tumoroids had less *Polg* expression than NT KD tumoroids (Fig. 5a). The *Polg* KD tumoroids also had less mtDNA content than NT tumoroids and similar mtDNA content as ETBF tumoroids (Fig. 5b). *Polg* KD tumoroids had significantly increased glucose uptake and lactate secretion relative to NT tumoroids, but less than ETBF tumoroids suggesting that, at least in part, the increased glucose uptake and lactate secretion in ETBF tumoroids is due to the promoter DNA methylation and reduced expression of *Polg* (Fig. 5c, d).

Mitochondria are major intracellular producers of reactive oxygen species (ROS) and are susceptible to oxidative damage [28]. They also play a key role in initiation and execution of apoptosis. Therefore, we hypothesized that tumoroids with fewer mitochondria would be more resistant to oxidative stress than those with normal levels. We determined that ETBF and *Polg* KD tumoroids are more resistant to oxidative stress than NT KD tumoroids by measuring cell viability 72 h after mock or 0.5 mM hydrogen peroxide (H_2_O_2_) treatment (Fig. 5d). Altogether, these findings demonstrate that KD of *Polg* reduces mtDNA content in both colonoids and tumoroids and increases glucose uptake, lactate secretion, and resistance to oxidative stress specifically in tumoroids.

## Discussion

It is well established that inflammation can induce silencing of genes by promoter CpG-island DNA hypermethylation, and this methylation, if it occurs in the promoters of tumor suppressor genes, can participate in tumorigenesis [29]. Tumorigenesis can also be initiated by mechanisms independent of chronic inflammation. Whether inflammation-driven DNA methylation events result in tumors that are phenotypically different from tumors whose formation is not directly from chronic inflammation is not well understood. Here, we uniquely demonstrate that inflammation-driven DNA methylation and gene silencing of *Polg* in murine colon tumors results in tumors with fewer mitochondria and altered metabolism in comparison to non-inflammation-induced tumors. Furthermore, tumoroids derived from these two types of tumors retain their DNA methylation, mitochondria, and metabolic differences. Using the tumoroids as a manipulatable system, we demonstrate that increased glucose uptake, lactate secretion, and resistance to oxidative stress in inflammation-induced colon tumors are, at least in part, due to decreased *Polg* expression.

Human colorectal cancer tissue has lower mtDNA copy number than surrounding normal tissue, and the copy number negatively correlates with disease progression [30–32]. There is also evidence of alterations in mitochondrial function in the epithelium of patients with inflammatory bowel disease, a condition that increases risk for developing colon cancer [33]. However, in these studies, which use human primary samples, it is unclear if decreased mtDNA is directly involved in disease progression or is a consequence of the disease itself. Here, we demonstrate that, in the setting of inflammation-induced tumors, decreases in mtDNA copy and in numbers of mitochondria are directly associated with increased glucose uptake, lactate secretion, and resistance to oxidative stress.

The cause-effect relationship between glycolysis and cancer is controversial. Mitochondria produce ATP through OXPHOS and synthesize key molecules needed for cellular proliferation. Some level of functional mitochondria and mtDNA is necessary for the survival of cancers cells [26]. However, dysfunction of mitochondria in tumor cells through mtDNA mutations and/or reduction in mtDNA copy number alters OXPHOS resulting in increased reliance on aerobic glycolysis [6]. Upregulation of glycolysis produces ATP to compensate for decreased ATP production by mitochondria. Glycolysis also provides intermediates for macromolecular biosynthesis and redox homeostasis [34]. These connections suggest that alterations in mitochondrial functions have a role in initiating or sustaining some tumors [6]. The loss of *Polg* expression in our system directly results in reduced mitochondria and a shift towards increased glycolysis. Importantly, the DNA methylation, reduced *Polg* expression, and decrease in number of mitochondria occur early in the tumorigenesis process (at the earliest time point when we can macroscopically detect tumors) and occur to some extent in all inflammation-induced wildtype MSH2 tumors, we have tested in our model. The strong selective pressure for this process suggests that it results in a survival benefit for cells that harbor DNA hypermethylation of the promoter of *Polg*.

Mitochondria are the major producer of intracellular ROS and reactive nitrogen species (RNS). Damage of mitochondria and impairment of mitochondrial respiratory function enhances ROS release into the cytoplasm [28]. Mitochondria also play a significant role in the induction of apoptosis [35]. While ROS can play a positive role in tumorigenesis, ROS levels above the antioxidant capability of the tumor cells are detrimental [36]. Having fewer mitochondria present in cells at sites of chronic inflammation would result in less mitochondrial damage and therefore potentially less intracellular ROS production and reduced induction of apoptosis. On the other hand, increased redox potential of ETBF Min tumors could account for the increased resistance to ROS. Even though glucose uptake is higher in ETBF than mock tumoroids, the levels of glucose in tumor tissues are not changed significantly suggesting that the additional glucose is rapidly metabolized. Only the upstream metabolite, G-6P, is higher in ETBF Min tumors. The levels of the TCA cycle intermediates and lactate are lower in ETBF Min tumors, and the levels of pyruvate are not changed. The glucose could be metabolized through the pentose phosphate pathway rather than glycolysis, which would result in production of the reducing agent NADPH (nicotinamide adenine dinucleotide phosphate) [34]. However, we found no differences in NADPH or reduced glutathione (GSH) levels between different tumor or tumoroid types (data not shown). While the exact mechanism of increased resistance to ROS needs to be further elucidated, we demonstrate that tumoroids with reduced expression of *Polg* (and consequently fewer mitochondria) are more resistant to oxidative stress suggesting a possible mechanism for the selective advantage for epigenetic silencing of *Polg* during chronic inflammation-induced tumorigenesis.

## Conclusions

*Polg* DNA methylation changes in inflammation-induced colon tumors make them metabolically different from non-inflammation-induced tumors and more resistant to oxidative stress. Understanding the metabolic differences and the role epigenetics plays in establishing and maintaining these differences is important for developing strategies to best treat cancers in patients that are caused by different etiologies. For example, pursuing differences in metabolism by using glycolytic inhibitors against cancer cells with mitochondrial defects may allow for targeted oncology therapeutics.

## Additional file

Additional file 1: **Figure S1.** Additional gene and protein expression in mock and ETBF-induced tumors. A) Gene expression by qPCR relative to mock epithelium from indicated tissue 8 weeks post-infection. Mean ± SEM. *N* = 6. *P < 0.05 compared with mock Min epithelium. ^#^*P* < 0.05 compared to *Msh2*^*l/l*^*VC*Min ETBF tumors. B) Western blots were run using protein isolated from indicated tissue 8 weeks post-infection. Blots are representative of two independent sets of biological replicates. **Figure S2.** *Polg* knockdown in colonoids derived from normal epithelium does not alter glucose uptake. A) *Polg* gene expression by qRT-PCR relative to nontarget (NT) knockdown colonoids derived from wt epithelium. Bar represents mean ± SEM. *P < 0.05. B) DNA from organoids as in A was used in qPCR assays with primer sets specific for a region of mitochondrial DNA or the genomic ApoB. Bars indicate mean ± SEM. *P < 0.05. C) Glucose uptake by tumoroids derived as in A. Mean ± SEM. NS not significant. **Table S1.** Primer sequences for qMSP and mtDNA content and assays used for TaqMan gene expression. **Table S2.** Fold change in expression by RT2 Profiler Glucose Metabolism PCR Array. (DOCX 3724 kb)

## Abbreviations

2-DG: 2-Deoxy-D-glucose
AKT: AKT serine/threonine kinase 1
Aldob: Aldolase, fructose-bisphosphate B
Apc: Adenomatous polyposis
EGF: Epidermal growth factor
ETBF: Enterotoxigenic *Bacteroides fragilis*
F2,6BP: Fructose-2,6-biphosphate
G-6P: Glucose-6-phosphate
Gck: Glucokinase
GC-MS: Gas chromatography-mass spectrometry
Glut-1: Glucose transporter 1
GSH: Reduced glutathione
H_2_O_2_: Hydrogen peroxide
HIF1α: *Hypoxia inducible factor 1 alpha*
HK: Hexokinase
KD: Knockdown
MBD-seq: Methyl CpG-binding domain sequencing
Min: Multiple intestinal neoplasia
MSH2: MutS Homolog 2
*Msh2*^*l/l*^*VC*Min: *Msh2*^*lox/lox*^*VillinCre*Min
mtDNA: Mitochondrial DNA
MTT: 3-(4,5-Dimethylthiazol-2-yl)-2,5-diphenyltetrazolium bromide)
NADPH: Nicotinamide adenine dinucleotide phosphate
NT: Nontarget
OXPHOS: Oxidative phosphorylation
PFK1: Phosphofructokinase 1
PFKFB: 6-Phophofructose-2-kinas/fructose2,6-biphosphatase
Polg: DNA polymerase gamma
Prps1l1: Phosphoribosyl pyrophosphate synthetase 1-Like 1
Pygm: Glycogen phosphorylase, muscle associated
qMSP: Quantitative methylation-specific PCR
RNS: Reactive nitrogen species
ROS: Reactive oxygen species
SEM: Standard error of the mean
TCA: Tricarboxylic acid
TCGA: The Cancer Genome Atlas
TEM: Transmission electron microscopy
