# Supplementary material for: Inflammation-induced DNA methylation of DNA polymerase gamma alters the metabolic profile of colon tumors
Source: Cancer Metab. 2018 Jul 10;6:9. doi: 10.1186/s40170-018-0182-7 (PMC6038244; doi:10.1186/s40170-018-0182-7)
Supplement: Supplementary file 1 — Figure S1. Additional gene and protein expression in mock and ETBF-induced tumors. A) Gene expression by qPCR relative to mock epithelium from indicated tissue 8 weeks post-infection. Mean ± SEM. N = 6. *P < 0.05 compared with mock Min epithelium. #P < 0.05 compared to Msh2l/lVCMin ETBF tumors. B) Western blots were run using protein isolated from indicated tissue 8 weeks post-infection. Blots are representative of two independent sets of biological replicates. Figure S2. Polg knockdown in colonoids derived from normal epithelium does not alter glucose uptake. A) Polg gene expression by qRT-PCR relative to nontarget (NT) knockdown colonoids derived from wt epithelium. Bar represents mean ± SEM. *P < 0.05. B) DNA from organoids as in A was used in qPCR assays with primer sets specific for a region of mitochondrial DNA or the genomic ApoB. Bars indicate mean ± SEM. *P < 0.05. C) Glucose uptake by tumoroids derived as in A. Mean ± SEM. NS not significant. Table S1. Primer sequences for qMSP and mtDNA content and assays used for TaqMan gene expression. Table S2. Fold change in expression by RT2 Profiler Glucose Metabolism PCR Array. (DOCX 3724 kb) [file 40170_2018_182_MOESM1_ESM.docx]

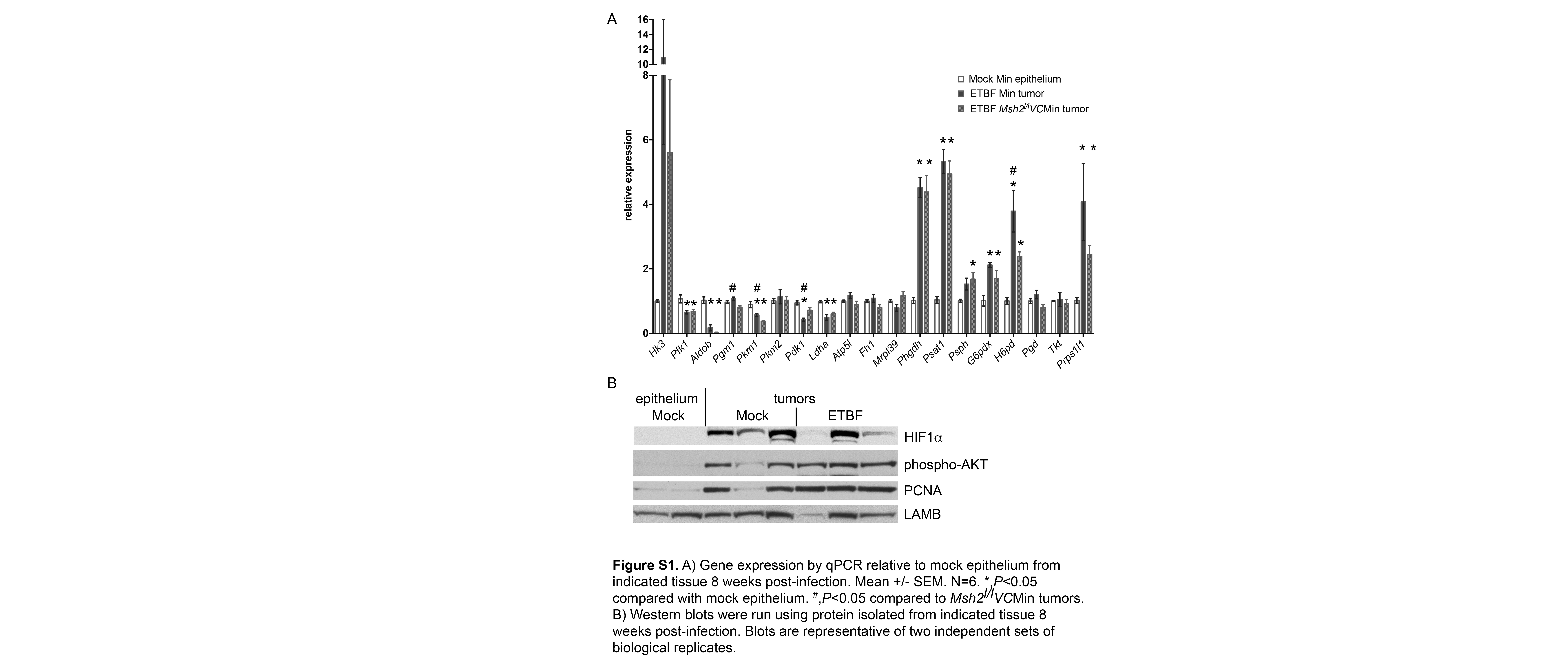


**Additinal file 1 Figure S1. Additional gene and protein expression in mock and ETBF-induced tumors.** A) Gene expression by qPCR relative to mock epithelium from indicated tissue 8 weeks post-infection. Mean +/- SEM. N=6. **P* < 0.05 compared with mock Min epithelium. ^#^*P* < 0.05 compared to *Msh2^l/l^VC*Min ETBF tumors. B) Western blots were run using protein isolated from indicated tissue 8 weeks post-infection. Blots are representative of two independent sets of biological replicates.


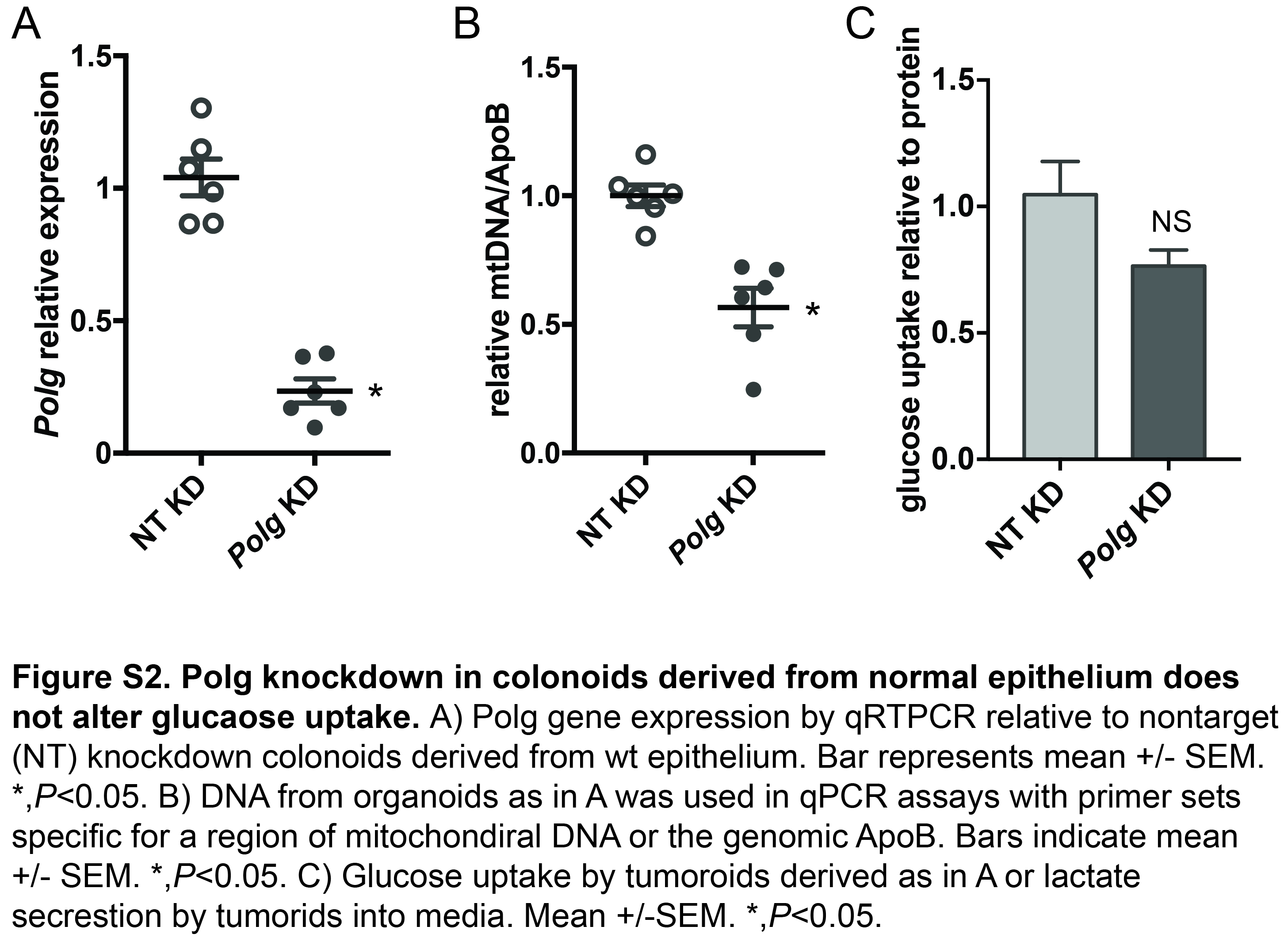


**Additional file 1 Figure S2. *Polg* knockdown in colonoids derived from normal epithelium does not alter glucose uptake.** A) *Polg* gene expression by qRTPCR relative to nontarget (NT) knockdown colonoids derived from wt epithelium. Bar represents mean +/- SEM. **P* < 0.05. B) DNA from organoids as in A was used in qPCR assays with primer sets specific for a region of mitochondiral DNA or the genomic ApoB. Bars indicate mean +/- SEM. **P* < 0.05. C) Glucose uptake by tumoroids derived as in A. Mean +/-SEM. NS – not significant.

| **Additional file 1 Table S1. Primer sequences for qMSP and mtDNA content and assays used for TaqMan gene expression.** | | | | | | | | | | |
| --- | --- | --- | --- | --- | --- | --- | --- | --- | --- | --- |
|  |  | |  | |  | | |  | | |
| **qMSP Primer Sequences** | | | |  | | |  | | |  |
| *Polg* | Left M | GTTCGTGTTTTTGTAGGTGTTTTAC | | Right M | | | CCCTCTACTCATCCAAATATTATCG | | |  |
|  | Left U | TTGTGTTTTTGTAGGTGTTTTATGT | | Right U | | | CCCTCTACTCATCCAAATATTATCAA | | |  |
| *Gapdh* | Left M | GTATTGTATAAGAAGATGCGGTCGT | | Right M | | | TACACGTAACTCAAACCTCTACGC | | |  |
|  | Left U | GGTATTGTATAAGAAGATGTGGTTGT | | Right U | | | TACACATAACTCAAACCTCTACACC | | |  |
|  |  |  | |  | | |  | | |  |
| **Applied Biosystems Taqman Gene Expression Assays** | | | |  | | |  | | |  |
| *Polg* | mm00450527_m1 | | |  | | |  | | |  |
| *PPIA* | Mm02342430_g1 | | |  | | |  | | |  |
|  |  |  | |  | | |  | | |  |
| **mtDNA content** | | | |  | | |  | | |  |
| mtDNA set1 | Fwd | AATCAATGGTTCAGGTCA | | Rev | | ACGGAGGATGGTAGATTA | | |  |  |
| mtDNA set2 | Fwd | CTCCGTGCTACCTAAACACCTTATC | | Rev | | GACCTAAGAAGATTGTGAAGTAGATGATG | | |  |  |
| ApoB | Fwd | CACGTGGGCTCCAGCATT | | Rev | | TCACCAGTCATTTCTGCCTTTG | | |  |  |

| **Additional file 1 Table S2. Fold change in expression by RT2 Profiler Glucose Metabolism PCR Array.** | |
| --- | --- |
|  | Fold change ETBF Min tumor relative to mock epithelium |
| **Prps1l1** | 20.39 |
| **Hk3** | 6.23 |
| Pygm | 2.6 |
| Rpia | 2.27 |
| **Gck** | -102.54 |
| **Aldob** | -19.16 |
| Phkg1 | -11.24 |
| Pck1 | -6.5 |
| Cs | -3.46 |
| Galm | -2.97 |
| Pdp2 | -2.93 |
| Suclg1 | -2.85 |
| Eno3 | -2.85 |
| Eno2 | -2.73 |
| Gapdh | -2.68 |
| Pdk1 | -2.48 |
| Idh3a | -2.46 |
| Suclg2 | -2.46 |
| Sdha | -2.33 |
| Pdpr | -2.31 |
| Aco1 | -2.28 |
| Gsk3a | -2.23 |
| Ogdh | -2.19 |
| Ugp2 | -2.11 |
| Idh3b | -2.07 |
| Prps1 | -2.04 |
|  |  |
| Changes in bold gene names were verified by qRTPCR. | |
